# Postcolonial theory and Canada’s health care professions: bridging the gap

**DOI:** 10.1007/s11019-021-10019-2

**Published:** 2021-05-12

**Authors:** Stephen Wilmot

**Affiliations:** Associate Academic, UDoL, University of Derby, Kedleston Road, Derby, DE22 1GB UK

**Keywords:** Canada, Postcolonial theory, Indigenous peoples, Paradigms, Ideologies, Legitimacy

## Abstract

In recent years there have been several calls in professional and academic journals for healthcare personnel in Canada to raise the profile of postcolonial theory as a theoretical and explanatory framework for their practice with Indigenous people. In this paper I explore some of the challenges that are likely to confront those healthcare personnel in engaging with postcolonial theory in a training context. I consider these challenges in relation to three areas of conflict. First I consider conflicts around paradigms of knowledge, wherein postcolonial theory operates from a different base from most professional knowledge in health care. Second I consider conflicts of ideology, wherein postcolonial theory is largely at odds with Canada’s political and popular cultures. And finally I consider issues around the question of Canada’s legitimacy, which postcolonial theory puts in doubt. I suggest ways in which these conflicts might be addressed and managed in the training context, and also identify potential positive outcomes that would be enabling for healthcare personnel, and might also contribute to an improvement in Canada’s relationship with its indigenous peoples.

## Introduction

In recent years several writers have argued that the perspective known as ‘postcolonial theory’ should be more widely adopted by Canadian settler healthcare personnel working with Indigenous people, to give a deeper historical understanding of the situation of Indigenous peoples in Canada. Those putting this argument include Anderson et al. (2003), Beavis et al. (2015), Browne et al. (2005), Holmes et al. (2008) and McGibbon et al. (2014). The significance of the postcolonial analysis lies in its identification of Canada as the product of expropriation and oppression of its Indigenous peoples. For the aforementioned commentators it is a necessary condition for Canadian healthcare professions to provide appropriate healthcare to Indigenous patients and clients that they understand the postcolonial account of Canada’s relationship to its Indigenous peoples. This would help combat prejudice and ignorance concerning Indigenous clients among settler personnel, and also challenge the appropriateness of specific policies and practices in healthcare for Indigenous clients and patients. Postcolonial theory already provides a philosophical base for the increasingly widely employed modality of cultural safety training but, it is argued, it needs wider recognition and a higher profile. In the light of that, there is a need to be clear about how the philosophical, ethical and political challenges of postcolonial theory can be managed. I propose to consider this.

Postcolonial theory was developed during the mid to late twentieth century by a number of writers, prominent among whom were Frantz Fanon (2008), Stuart Hall (2017), Edward Said (1978) and Gayatri Spivak (1999). They developed an analysis of colonialism in its impact on the colonized in the Middle East, the West Indies and South Asia, but the theory has also been applied to settler-dominated colonized Indigenous minorities, particularly in North America and Australasia. In the postcolonial analysis colonialism is rooted in economic exploitation which is normalized or concealed in the dominant discourses of colonial societies. The impact of this exploitation on colonized peoples is overwhelmingly negative, running from cultural/psychological disempowerment to genocide. In terms of its philosophical affiliation, Holmes et al. (2008) argue that postcolonial theory is rooted in critical theory, which links it, inter alia, to Marxism and poststructuralism.

A brief historical account is relevant here. Over 5 centuries Canada has developed from the colonization of northern North America by France and Britain, the occupation of the country by settlers from those countries, and the claiming by them (later by Britain exclusively) of the colonized territories. Indigenous peoples were required to cede land and independence and become subjects of the colonial powers. Canada became independent from Britain by stages during the nineteenth and twentieth centuries, and at present Indigenous peoples form around 4.9% of the population (Statistics Canada 2017). They are significantly worse off than the rest of Canada in terms of income, education, housing, and health (Indigenous and Northern Affairs Canada 1996; Health Canada 2014). Part of this disparity can be attributed to the failure of the healthcare system to provide appropriate care, exacerbated by lack of attention to the social determinants of health (Health Council of Canada 2012). Attempts by Canadian authorities to intervene in Indigenous life, often intended to accelerate cultural assimilation, have typically proven very disruptive to Indigenous families and communities. Indigenous children were subjected to compulsory residential schooling for many years, and there has also been a disproportionate rate of child removal from Indigenous families with damaging consequences to their development (Truth and Reconciliation Commission of Canada 2015). Attempts by Canadian governments and other organizations to undo some of this damage in recent decades has been accompanied by the development of an Indigenous program of demands for a re-evaluation of Canada’s relationship with its Indigenous peoples (Idle No More 2012; Marshall 2019). Postcolonial theory is relevant to all these processes.

The question I propose to explore is as follows: how can we optimize engagement with, and application of, postcolonial theory by those practitioners who are accustomed to the dominant settler liberal individualist culture that is fundamentally at odds with postcolonial theory*?* My focus is on settler health care personnel, as they constitute the overwhelming majority of the healthcare workforce. One estimate suggests that only 1.2% of Canadian health care practitioners are Indigenous (University of Saskatchewan 2017), and in such numbers the prospect of Indigenous personnel permeating change in the attitudes of settler colleagues is very limited. Rather, bringing about this change is a task for the healthcare system as a whole in the context of a wider anti-oppression agenda, while ensuring responsiveness to the perspectives of Indigenous colleagues.

I intend to explore the requirements and challenges of the postcolonial analysis for those personnel, and the ways in which it might achieve a positive impact in the context of a settler liberal state and political culture. I shall work from the assumption that relevant personnel will be at different positions on a continuum in terms of their awareness of the issues raised by postcolonial theory but that all would benefit from some training in its principles and application. And I shall consider how its application might be managed in the training. I shall do this on the assumption that settler practitioners who undergo postcolonial training will vary in their responses; and that they will not necessarily all embrace the postcolonial worldview enthusiastically, but that they will nonetheless take seriously the implications of its propositions and attempt to apply these to practice situations.

As stated earlier, my analysis will be philosophical, ethical and political. I shall look first at the implications of postcolonial training for the knowledge-bases of health and social care practice. I shall then consider its implications for the wider belief-systems of practitioners who engage with it. Finally I shall consider its impact on the practitioners’ relationship with their country.

### Knowledge-base

Professions in healthcare have a wide range of theoretical bases, and the influence of a particular theory in any given profession will be affected by a number of factors, including the power and influence of key actors both within that profession and outside; and also by the power structure within organizations, professional training institutions, universities, research bodies and academic publications. Mackey (2007) offers a Foucauldian analysis of some of these factors that locate power and dominant discourses in a profession. The widely dominant discourse of evidence-based practice requires that the application of a particular theory in healthcare be supported by research evidence, and the criteria by which that evidence is judged is central to this analysis. A crucial guiding concept in identifying these dominant criteria is that of research paradigms, and I shall use the formulation of these paradigms developed by Guba and Lincoln (1994) to analyze this further. Guba and Lincoln argue that the credibility of research is established predominantly within the terms of one of four research paradigms- positivism, post-positivism, constructivism and critical theory, representing three alterative epistemologies. Positivism has traditionally been the epistemology of the physical sciences, but has also been used in sociology, psychology and other social sciences. It posits an objective, quantifiable and predictable reality, favouring evidence from quantitative research. According to Guba and Lincoln it has been somewhat superseded in some disciplines by post-positivism, a development of positivism that acknowledges a greater degree of subjectivity and is more accommodating of qualitative research evidence. By contrast the third paradigm, constructivism, posits a subjective, negotiable, and contingent reality to be understood in concrete specifics, drawing predominantly on qualitative research evidence. It is influential in parts of the social sciences.

However, postcolonial theory is located in the fourth of the paradigms, critical theory, so I shall now focus on this. Critical theory includes Marxism in its philosophical ancestry, and is to varying degrees cognate with poststructuralism, and with feminism and other emancipatory ideologies. Postcolonial theory sits within this conceptual framework, with a specific focus on the oppression of colonized peoples by the forces of colonialism, which is itself a manifestation of capitalism. So it is important for practitioners engaging with postcolonial theory to do so in the context of critical theory.

Critical theory operates to rather different principles from the other three paradigms mentioned above. First, its ontology is fundamentally different from those of the other three. Guba and Lincoln ascribe ontologies—accounts of the nature of reality- to each paradigm. Positivism is naïve realist, postpositivism is critical realist, constructivism is relativist and critical theory is historical realist. Ontologies define the categories of knowledge that can be obtained by the investigator—what is knowable in the world. The ontology of critical theory differs fundamentally from the other three, in that it includes a general theory of politics and history centring around the proposition that inequality and oppression are structured into western society and culture, and all aspects of knowledge need to be seen as produced by those processes. The other ontologies offer no equivalent analysis.

Critical theory is also fundamentally different from the other paradigms in its epistemology. That manifests in two ways. First, critical theory views the investigator very differently from the others. For positivists and to a lesser degree postpositivists, the investigator is objective and detached, standing outside the field to be investigated, while for constructivists the investigator is open, involved and subjective, constructing the field as they investigate it. But for the critical theorist the investigator, whether they know it or not, and whether they choose to be or not, is fully involved in, and contaminated by, the power dynamics defined in its ontology. So the knowledge obtained is also defined and shaped by those political realities*.* Critical theory frames knowledge as a product of political struggles between oppressors and oppressed; and valid knowledge as derived from understandings gained by the investigator through a dialectical process of observing and reflecting on the conflictual dynamics of society, economy and state. The concept of critical consciousness is typically applied to this understanding of the way the world works, and posits the gaining of insight into those dynamics; dynamics that are disguised and obfuscated by dominant discourses in the societies concerned (see for instance Getzlaf and Osborne 2010).

The second difference concerns the starting-point of investigation. Whereas the other paradigms focus initially on segments of reality, and seek to build a bigger picture from these segments through research (the segments might be quantitative or qualitative) critical theory starts with the whole picture. It is primarily concerned with understanding the context of data, without which the data itself is meaningless—a principle inherited from Marx and Hegel. And it can include other paradigms in its context, so attaining a ‘meta’ vantage-point in relation to them. This facilitates a critical theory critique of the other paradigms, and in this respect positivism and postpositivism receive particular attention, being characterized by critical theorists as dominant and oppressive, and as denying, legitimizing or concealing inequalities of power. Holmes et al. (2008) offer an example wherein general critical theory principles in the format of postcolonial theory are applied to a critique of positivist and postpositivist dominance in the context of nursing. In this way also, postcolonial theory opens the way to a political critique of research methods in the health care professions, and of their research culture and ethics.

So the relationship between critical theory and other paradigms is asymmetrical. Positivism, postpositivism and constructivism have no equivalent critique of critical theory. Nor do any of the theories supported within those paradigms have an equivalent critique of postcolonial theory. So it is not possible to treat them as equivalents, or to approach their conflicts as resolvable in a comparative way.

There is one more consequence of the contextual focus of critical theory. The logic of adopting a critical-theory-based perspective like postcolonial theory is quite different from the logic of adopting a theory validated by evidence within the positivist or post-positivist paradigm. For a practitioner operating in one of the other paradigms to accept and adopt a particular theory-based intervention they would require evidence that supports the effectiveness of that intervention. The evidence may be quantitative or qualitative or both, but it would need to be in incremental quantities, additive to what the practitioner already knows. By contrast, adoption of an intervention within critical theory would need to be achieved initially through a reinterpretation- a ‘re-contextualizing’- of the existing body of knowledge. Although that process may also draw on new information, what matters is the reinterpretation and re-contextualization of the available information. Concepts such as consciousness-raising, and conscientization are specifically applicable here, in the tradition of commentators such as Freire (2017). Getzlaf and Osborne (2010) offer a practical application of this principle in professional education.

These features of critical theory have implications for the ways in which practitioners might compare and evaluate the theories in this frame, and make decisions about applying them. Because it combines an account of the world and a theory of knowledge, critical theory is both a theory of politics and history, and an epistemology. Its theoretical effectiveness can only be evaluated within the terms of its own epistemology, which depends on its ontology, which is that theory. The question of whether we can decide that critical theory makes sense in application can only be addressed within the terms of critical theory itself, because the evidence supporting the theory can only have validity within its own terms. In order to establish whether it works, we need to have embraced it already. And embracing it presents practitioners with an apparently all-or-nothing choice. They will be faced with a conflict between the requirements of the theories, research, and evidence-base that account for (in many cases) all of their practice up until that point, on the one hand; and the requirements of postcolonial theory on the other. The two cannot be combined or reconciled. They will have to coexist in practice if the benefits of postcolonial theory are to be brought into the practice of healthcare, but the juxtaposition will be uncomfortable for those who are engaging with and applying both.

There is no easy way of managing this conflict, but the relationship between the paradigms needs to be addressed in training. The better the practitioners understand the conflicting propositions of the paradigms, the more likely they will be to manage and mobilize the constituent ideas to enhance their practice, and learn to navigate the contradictions between them. Guba and Lincoln recommend ‘continuing dialogue among paradigm proponents’ (p 116) while Mesel (2013) argues for transparency of paradigms, enabling paradigms to be used explicitly and co-operatively. Different professions and disciplines will doubtless respond differently to this dialogue. Nursing has a history of exploring the four paradigms in some sectors of advanced training and research. Some social work training draws on critical theory. But some other professions are more limited in respect of the range of paradigms. The mapping of the paradigms will require a focus on the conflict between them and the process whereby critical theory deconstructs the others, so a good deal of conceptual exploration will be needed here. Also, critical theory is essentially (like its ancestor Marxism) a conflict theory, and in a culture that values consensus, management of this feature would require a significant adjustment by learners, and a good deal of skill on the part of trainers.

However, some common ground exists, and this can provide a smoother passage for practitioners in certain areas. This is particularly so in the area of research epistemologies and methodologies in healthcare. As part of its general critique of colonialism, postcolonialism offers a political rationale for serious engagement with Indigenous knowledge and Indigenous philosophies, and as part of the same agenda it also advocates egalitarian, collaborative research methods, allowing non-oppressive research initiatives with oppressed groups. As an example Edwards and Brannelly (2017) overview a range of methodologies for democratizing research, and include decolonizing/indigenous methodologies among these. Similarly Browne et al. (2005) and Willows (2019) advocate a radical rebalancing of power in the research process wherever issues relevant to Indigenous people are addressed; and, as part of this, advocate the inclusion of Indigenous ways of knowing in the research process. Their argument is that that while Indigenous peoples have knowledge and wisdom that is of value to the wider world, they also often have distinctive ways of organizing and validating knowledge which, in healthcare research, shapes how Indigenous people’s perspectives and experiences should be understood. This is also explored and illustrated by Barwin et al. (2013).

I would suggest that the above arguments have some common ground with more mainstream perspectives. For instance points related to those above are made by exponents of global bioethics such as ten Have (2016), who argues that the particular vulnerability of Indigenous peoples (among other groups) justifies specific measures in relation to research ethics to ensure full sharing of research-related power and benefits with those groups. There is also some common ground with Guba and Lincoln’s constructivist perspective, which likewise values non-dominant forms of knowledge and knowledge-gathering. So, ideas from global bioethics and from constructivism can both to some degree offer professionals a gateway to the postcolonial account of colonization and to Indigenous views of health, though without fully encompassing postcolonial political analysis. So those influenced by research bioethics may find common ground with postcolonialism through broader ethico-political arguments, while constructivists can argue epistemological rather than political justifications in a framework of cultural relativism. The fact that they arrive at common goals provides a bridge between the perspectives.

There is also another way in which postcolonialism might offer something to practitioners of other persuasions. Research in health care is typically (though not always) organized in a somewhat hierarchical way, particularly in academe, and practitioners in the field can experience this as excluding. Critical theory and postcolonialism contribute strongly to a wider rationale for democratizing that system, so the critique offered by critical theory is likely to be experienced as empowering for many professionals. In this way, the necessary addressing of ideological conflicts can be made less forbidding, and postcolonialism can be seen as having something to offer to practitioners.

On a practical note, the kind of intellectual endeavor I have just described normally happens in universities, but for these purposes I would suggest that it needs to come out into the training programs of service delivery organizations, and be facilitated by training personnel experienced in in-service training. The learning should ideally be team-based, and if that is not possible, at least to be clearly identified with and owned by the service-providing and employing organization. And as far as possible it needs to be interprofessional. Cross-fertilization of ideas and interpretations between different professional cultures and knowledge-bases would greatly facilitate the learning process, by foregrounding and questioning practices and ideas that might normally be taken for granted.

### Belief-systems and the community

Postcolonial theory raises many questions about Canadian society, and its impact cannot be confined to professional practice. We also need to consider how postcolonial theory might impact on the relationships of settler healthcare practitioners as citizens, community members and colleagues. In terms of those relationships it is likely that adopting the perspectives of postcolonial theory with regard to Indigenous peoples would distance settler practitioners somewhat from the views of many of their compatriots. There is a good deal of evidence that negative stereotypes of Indigenous people are still widespread among settler Canadians, and that arguments drawing on postcolonial theory are unpopular. Let three examples suffice. First, a nationwide survey by Environics (2016) found that widespread stereotyping of Indigenous peoples persists among ordinary Canadians. Second, Denis (2015) studied the detailed processes of white assumptions of superiority to Indigenous people and found that that process is still a significant shaper of attitudes in his sample from small-town Canada. Third, Baker and Verrelli (2017) found that Canada’s media continue to marginalize Indigenous political agendas. So if practitioners engage seriously with postcolonial principles and implications, that will distance them somewhat from the above attitudes and from the Canadian ideological mainstream.

According to Cochrane (2010) Canadian society accommodates a wide range of philosophies and ideologies in its population, so we may wonder why postcolonial theory should raise more difficulties than any other position on that spectrum. However, there are features of the postcolonial perspective that present specific challenges, and I shall explore those in the context of a particularly relevant piece of political theory, John Rawls’ model of political deliberation in a liberal democracy (Rawls 1993). Rawls’ framework is well suited to Canada’s pluralist liberal society embodied in Canada’s ideology of multiculturalism (Semotiuk 2017). Rawls’ thesis is that it is possible to separate broad personal and communal belief-systems from more specific political principles relating to governance and justice, and to do this to such a degree that people of differing ‘reasonable’ belief-systems can still achieve a workable degree of consensus in the sphere of politics and justice. He called the wider social and moral belief-systems ‘comprehensive doctrines of the good’. Religious belief-systems feature prominently among the examples he gave, but Kantian liberalism was also included**.** The label of reasonableness conferred by Rawls on some belief-systems is crucial here, and his definition of reasonableness includes at least two parts. First, reasonable doctrines do not require their adherents to coercively impose them on others. Reasonable Catholicism proposes that non-Catholics are wrong about certain major matters, but that does not require Catholics to coerce their fellow-citizens to change their views. Second, reasonableness in Rawls’ view also comes partly from the recognition by adherents of these doctrines that there exists what he called the ‘burdens of judgment’—that is, the imperfection of all human reasoning, and the question-mark that places over our adherence to our chosen doctrine, and over our rejection of other doctrines. That recognition enjoins tolerance, even respect, for those who disagree with us. If these doctrines are ‘reasonable’ then the political matters that need some degree of consensus in a well-run democracy could be agreed on through what Rawls called ‘overlapping consensus’ whereby basic principles- for instance equality before the law- would fit with all reasonable comprehensive doctrines, albeit in different ways and for different reasons.

The postcolonial perspective can usefully be viewed through the prism of Rawls’ ideas. It is inclusive enough to provide a doctrine of the good- not as fully comprehensive as for instance Roman Catholicism or liberalism, but nonetheless constituting an instance of what Rawls termed a ‘partially comprehensive doctrine of the good’ (Suissa 2010 p 591). Its parent-philosophy, critical theory, has something to say about most matters and offers a viable alternative to competing comprehensive doctrines of the good, religious or philosophical. Postcolonial theory is clearly a reasonable doctrine (by Rawls definition) on the first count mentioned above, in that it does not advocate the coercion of non-adherents. However, on the second count, concerning the burdens of judgement, its approach to those who reject it and/or adhere to another doctrine is quite unlike other reasonable doctrines. The other doctrines do not, on the whole, give much attention to explaining why some people do not adhere to them. Catholicism includes some explanations for the error of unbelievers, though these seem to be deployed sparingly in most situations. Liberals on the whole barely concern themselves with the matter. By contrast, for postcolonial theorists, and indeed for critical theorists generally, explaining and contextualizing the resistance of non-adherents is an important part of the theory. This feature is part of the Marxist heritage, elaborated by Gramsci, who formulated an explanation for rejection of Marxism centring on what he termed ‘hegemony’—the creation of a culture of false consciousness by capitalism, disguising oppression and inequality (Anderson 1976). Critical theory and postcolonial theory follow this tradition, giving central importance to the ways people are deceived by a hegemonic cultural system. This leaves no space for Rawls’ concept of the burdens of judgment as an account of opposing views or for the kind of mutually respectful discussion that Rawls envisages for a pluralist liberal polity.

These features are likely to create a tension between postcolonialists and many of their fellow-citizens who have different doctrines of the good, as the logic of postcolonial theory conflicts with Canada’s consensus-oriented culture. Where consensus is the norm, but disagreements come into the open, those involved may be unnerved by such disagreements. And if some or all of the protagonists are unclear about the assumptions that either they or their opponents are working from, this will lead to confusion as to why they are disagreeing. Given a widespread predisposition to formulate one’s own explanations of others’ behaviour in the absence of shared explanations, the danger is that those ‘do it yourself’ explanations will be based on stereotypes. Mock and Homer-Dixon (2015) theorize helpfully about this process. The worst outcome would be for those involved to conclude that postcolonial theory is too explosive to be workable. If it is to be applied, a way through this problem needs to be found.

As a response to this, postcolonial training needs to offer a ‘roadmap’ of doctrines of the good (using the Rawlsian term), to help participants clarify where they and others are coming from in ideological terms. This would give participants a sense of control over the dialogue, and understand the significance of what those who disagree with them are saying to them. An intellectual framework for dealing with the ideological interface would need to be offered. There are several theories of ideological and moral pluralism in a liberal society, but Rawls’ would seem a likely candidate to be used to provide a framework for identifying the features of ideological disagreement. This would help participants to explore the relevant ideological principles and arguments, and to understand how conflicting ideas might be accommodated in an open society, and especially in the case of postcolonial theory, to understand the concepts of hegemony and false consciousness.

Clearly doctrines and ideologies cannot be left as unexplored ‘givens’ in this situation. Those involved need to consider their own and others’ routes toward the particular ideologies and doctrines that have influenced their thinking. One of the crucial insights of critical theory is that doctrines and ideologies are produced through political and social as well as psychological processes. There are other perspectives that identify social and psychological routes taken by people to arrive at their particular philosophies, including those of Lakoff (2002) and Haidt (2013), and it is important that protagonists have some appreciation of those routes. This approach, hopefully, would allow recognition of the burdens of judgement in the liberal framework to coexist with understanding the politics of ideology in the critical framework.

Professional codes of ethics would inevitably come into question through this training process. These are likely to be a focus of debate, but it is fair to say also that exploring this area may offer something useful to practitioners in the learning process. One focus of debate might arise from the fact that Critical theory does not accept the separation of the ethical realm from other realms of knowledge. This separation characterizes parts of the western ethics tradition, and is well exemplified by the Principlist approach to health care ethics, based the four principles of beneficence, non-maleficence, justice and autonomy, which some commentators (notably Beauchamp and Childress 2019) see as standing independently of wider ideologies and ethical doctrines. That is particularly at odds with the critical theory view which is that ethics is embedded in practice and power, a view that is likely to resonate for many practitioners whose experience of dealing with ethical issues is often embedded in exactly those dimensions. Again, the critical theory approach may find common ground with perspectives such as the global bioethics approach advocated by ten Have (2016), focused on the values of solidarity and co-operation.

Legitimizing and exploring this more politically situated view of ethics also opens the door for learners to approach Indigenous ethical traditions—for instance as presented in overview by Borrows (2019)—not as curiosities of multiculturalism, but as expressions of a dynamic world-view that merits full and equal recognition and engagement. It also provides a way into the content, structures and assumptions of those Indigenous traditions themselves. For instance, Some Indigenous traditions frame health as a collective as much as an individual good, a view that has considerable ethical implications for community members’ responsibilities to one another, and which resonates, again, with the global bioethics values of solidarity and co-operation. Also in many Indigenous traditions, spirituality is a crucial dimension to moral thinking in healthcare (see for instance Levesque et al. 2013). These and other insights are likely to be a significant reinforcement for practitioners in engaging with differences of perspective- reinforcing, that is, the sense that they are learning something that is usable in practice.

In these ways practitioners in the training process can be helped to work across the ideological boundary without disabling conflict, and to discover real potential rewards in doing so. I suggested earlier that participants in the training situation need to take postcolonial theory on board to a significant degree if it is to achieve any improvement in services to Indigenous patients. However there also needs to be acknowledgement that this process is not necessarily indicative of its absolute truth, but rather that it is contingent, and in particular contingent on its usefulness in helping to justify outcomes that accord with more widely-held moral principles of justice and equity—hopefully principles that the participants themselves can accept. There is a danger otherwise that practitioners come to see postcolonial theory as a zero-sum ideology that takes no prisoners. This would be a major disincentive for many practitioners to taking it seriously in the professional context.

### The legitimacy of Canada

Thus far I have identified potential challenges arising from the promotion of postcolonial theory, and suggested ways of managing them through training. However, for this third section I propose to take a different approach, to consider the matter initially as an opportunity with major possibilities; and to focus on the way postcolonial training might optimize those possibilities. I propose to consider the implications of postcolonial theory for the settler practitioners’ relationship with their country, particularly in relation to its deconstruction of Canada’s legitimacy as a state. Citizens of a stable liberal democracy like Canada will need compelling reasons to doubt the legitimacy of their country, and from the viewpoint of the individual citizen, there are strong psychological and social reasons to accept its legitimacy as the default position. However, among those Canadians who have embraced the postcolonial perspective on Canada’s history, some might well have already come to see Canada’s right to their loyalty as contingent and questionable, on the basis of that perspective, for at least two reasons. First, Canada’s creation involved the expropriation of Indigenous peoples without their consent, and this creates a moral fault-line in Canada’s foundations. In the view of a number of writers, including Buchanan (1999) and Ivison (2017), such a foundational fault undermines a state’s legitimacy. Second, Canada now rules without consent over those same Indigenous peoples, thus losing some legitimacy in relation to that section of its citizens, and to others who identify with them.

So how do positive possibilities arise from this? The Canadian state stands in a very different place in the relationship between the healthcare system and Indigenous people, when compared to the same healthcare system’s relationship with most settler Canadians*.* In the eyes of the latter the healthcare system has a base of legitimacy that derives partly from the professional status of its practitioners, partly from the organization. Both of these identifications depend in part on the fact that they are located in specific national and provincial systems of healthcare provision and professional regulation. The Canadian state constitutes the ground that the healthcare system stands on. Its credibility in the eyes of the majority of service-users, and the right of its practitioners to do what they are doing, are underpinned by the legitimacy of the Canadian state. Whatever the contingencies of the transaction and the relationship, that legitimacy provides a bedrock of mutual recognition and trust.

For Indigenous peoples there is no such underpinning. And to consider why, I need to look briefly at theories of legitimacy. According to Peter (2017) the main theories in this field focus on the political process of state development and policymaking (for instance, Lockean principles of consent and Rawlsian principles of political reasoning) or on the beneficial outcome of state and government policy (for instance Millian utilitarianism). The Canadian state’s record on all of these counts in relation to Indigenous healthcare is seriously compromised. State healthcare provision has in many respects been an alien imposition (see for instance Kelm 1998; Drees 2010) and has thereby commanded insufficient Indigenous consent, and represented insufficient political reasoning involving Indigenous peoples (Alfred and Corntassel 2005). And in its failures regarding Indigenous health it produces insufficient utility for Indigenous peoples (see for instance Health Council of Canada 2012). The history of service provision to Indigenous peoples includes harms that did not occur by mischance but through the application of colonialist ideology. Canada’s healthcare system is tainted by the fact that it has been an integral part of what has been experienced by Indigenous peoples as an assault on culture, family, community and identity. Overall Indigenous peoples have experienced the Canadian state in a way quite different from other Canadians, and in Shaw’s words, they ‘know there are dominant systems and forms of power. They know what it feels like to be victim to them; they know these systems work for others; they know, and have borne, some of their costs’. (Shaw 2008 p 5). That is not, on the whole, the experience of settler Canadians.

So the above-discussed criteria for trust and legitimacy are not met, and thus the Canadian state cannot confer legitimacy on the actions of social or healthcare personnel. Nonetheless Canada is still very much present in the transaction, in terms of resources and machinery of provision, as its obligations to provide services to Indigenous peoples are still in effect, and need to be implemented on the ground. This places a very specific demand on healthcare personnel in this situation. The (largely) unquestioned, unexamined moral base of Canada that is the context for relationships with most settler Canadian clients cannot apply in work with Indigenous clients; it must be replaced by an alternative principle for legitimizing healthcare provision, one that acknowledges the harm of colonialism. In work with Indigenous service users, settler healthcare personnel need to achieve a level of trust that can be sustained outside of Canada’s claim to legitimacy, so postcolonial training needs to enable them to work without the legitimizing ground of Canada to stand on. The postcolonial perspective provides a basis for this, in the concept of settler solidarity and alliance with Indigenous people in combating colonialism, as explored by Bacon (2017) Morris (2017) and Nixon (2019).). This specific solidarity, again, can be linked to the wider bioethical value of solidarity (ten Have 2016). The training needs to make that operational.

That solidarity requires a supporting principle that replaces the state in legitimizing the relationship. A possibility would be contractarianism. The principle of contract as a moral framework accommodates some of the necessary features that legitimize what the state cannot itself legitimize. Ideally contracts are consensual and result from reasoned negotiation. They bind the parties in specific terms with clear limits but maintain a degree of distance and rationality that (in this case) provides some safety to Indigenous communities and clients. They are not proof against racism, oppression or inequality, but they do militate against these by limiting their effects, for instance by minimizing the presence of unspoken assumptions in negotiations and decisions. They also avoid ‘mission creep’ by placing limits on the degree to which interventions can extend beyond original needs and plans. However the most important characteristic is that they provide a moral framework that is explicit, documentable and independent of the state; and in this way create a structure to contain the process of service provision and the relationship between provider and recipient, and to legitimize the accountability of the personnel involved (see Wilmot 1998 for further discussion of contractarianism in healthcare).

Postcolonial training for a contractarian relationship would need to enable personnel to navigate the general principles of the contract, and at the same time think creatively about the kind of arrangements that might make the contractual relationship work. I have mentioned some general principles, but a number of questions arise with regard to the practical arrangements. For instance, who would be the parties to the contract? The requirements of possible parties- individual practitioners, teams, agencies, Indigenous communities, Indigenous agencies and governments—would need to be explored, with a view to understanding possible relationships between them. Professional ethics and legal requirements would need to be considered. Practitioners would need to develop skills of creative thinking in identifying potential relationships and establishing goals.

Earlier I suggested that accepting the legitimacy of one’s country is a default position for most settler Canadians. Since then I have skated over the issue of likely discomfort involved for healthcare personnel in having that legitimacy undermined by postcolonial training. Focusing on it now, the first requirement is that such discomfort be understood positively as an effect of growth and progress. Group support can facilitate this, but perhaps the most powerful reframing of this discomfort can be constructed around the final goal of the training. Each practitioner who successfully undergoes training will thereby be equipped to contribute to changing Canada’s treatment of Indigenous peoples in a way that brings a wider settlement with those Indigenous peoples- and therefore an agreed Canada- a little closer. In this way everyone involved in the training can see their way forward to helping heal Canada’s legitimacy deficit.

## Conclusion

I have sought to map out three likely challenges (and opportunities) presented by postcolonial theory in training, and to suggest ways in which those might be addressed, to ensure that personnel who undertake this are equipped to manage its impact in the most effective way. The level is somewhat theoretical and speculative, as there is little evidence so far on the actual effect of postcolonial training in this context. But given the progress of this approach, my comments will, I hope, provide some helpful thoughts. Postcolonial theory provides a radical analysis of relationships in a settler-colonial society and state and by virtue of that, there is a chasm between it and Canada’s political culture; first because of its radical historical critique of Canada’s existence, and second because of its basic incompatibility with the liberal free-market nature of the aforementioned political culture. My comments are geared primarily to attempting to bridge the chasm in a way that acknowledges the incompatibilities while finding concrete ways to work across them.
